# Dysregulation of microRNAs and Centromere Protein Genes in Prostate Cancer and Metastatic Progression

**DOI:** 10.5152/tud.2026.26143

**Published:** 2026-04-02

**Authors:** Makbule Nihan Somuncu, İlknur Karalezli, Yunus Emre Göger, Ayşe Gül Zamani, Mahmut Selman Yıldırım, Asuman Deveci, Mustafa Cihat Avunduk, Giray Karalezli

**Affiliations:** 1 Department of Medical Genetics, Necmettin Erbakan University Faculty of Medicine, Konya, Türkiye; 2 Selçuk University, Vocational School of Health Services, Konya, Türkiye; 3 Department of Urology, Necmettin Erbakan University Faculty of Medicine, Konya, Türkiye; 4 Sakarya University Faculty of Medicine, Sakarya, Türkiye; 5 Department of Pathology, Necmettin Erbakan University Faculty of Medicine, Konya, Türkiye; 6 Department of Urology, Beyhekim State Hospital, Konya, Türkiye

**Keywords:** Biomarker, cell cycle, metastasis, miRNA, prostate cancer

## Abstract

**Objective:**

Accurate prediction of disease progression and metastatic potential remains a major challenge in prostate cancer management. This study aimed to evaluate the expression profiles of selected microRNAs (miRNAs) and cell cycle–related genes as centromere protein genes (CENPs) in prostate cancer and to explore their potential clinical relevance in distinguishing metastatic disease.

**Methods:**

Formalin-fixed paraffin-embedded prostate tissue samples from patients with benign prostatic hyperplasia, localized prostate cancer, and metastatic prostate cancer were analyzed. Quantitative real-time polymerase chain reaction (PCR) (qRT-PCR) was used to assess the expression of miRNAs targeted CENPs as well as CENPA,CENPC, HJURP,andAURKB. Expression patterns were further validated in the metastatic prostate cancer cell line PC3. Relative expression levels were calculated using the 2^−^ΔΔCt (cycle threshold) method.

**Results:**

Expression analysis of selected miRNAs revealed a significant upregulation of hsa-miR-4755-5p in the metastatic PCa (*P* = .027), despite no significant differences in fold change. Additionally, hsa-miR-5680 showed a significantly higher fold change in the metastatic group compared to controls (*P* = .034). Although not statistically significant (*P* > .05), hsa-miR-5688, hsa-miR-20a-5p, and hsa-miR-4500 demonstrated a consistent trend toward downregulation, as indicated by lower ∆∆Ct values in both PCa and metastatic groups relative to controls. In PCa cell line CENPA, CENPC,andHJURP expression levels increased over time, whereas *AURKB *was downregulated.

**Conclusion:**

The findings indicate that combined dysregulation of specific microRNAs and centromere-associated genes characterizes metastatic prostate cancer. This molecular signature may provide clinically relevant information for risk stratification and warrants further investigation as a potential adjunct to conventional prognostic markers in prostate cancer.

**Main Points**

- Metastatic prostate cancer exhibits a distinct pattern of microRNA (miRNA) dysregulation together with altered expression of centromere-associated genes.
- hsa-miR-4755-5p and hsa-miR-5680 are significantly upregulated, while hsa-miR-5688, hsa-miR-20a-5p, and hsa-miR-3941 show a consistent downregulation trend in metastatic disease.
- CENPA, CENPC, and HJURP expression is increased, whereas AURKB shows cell line–specific downregulation in PC3 cells.
- Concordant findings obtained from clinical tissue samples and prostate cancer cell line models support the translational relevance of the study.
- Combined miRNA and centromere gene expression profiling may contribute to improved risk stratification in prostate cancer.

## Introduction

Prostate cancer (PCa) remains one of the most frequently diagnosed malignancies and a leading cause of cancer-related mortality among men. Recent data report around 1.47 million new cases and over 375 000 deaths annually.^1^ By 2040, incidence is projected to reach 2.9 million and mortality over 700 000, highlighting the urgent need for improved prevention, diagnosis, and treatment.^2^ PCa exhibits marked heterogeneity, from indolent tumors to aggressive, metastatic forms, with prognosis strongly influenced by tumor stage, Gleason score, and metastasis.^3^

Recent advances in PCa diagnostics have highlighted molecular imaging and tissue biomarkers to improve accuracy and personalize treatment. However, despite advances in early detection through prostate-specific antigen screening or improvements in techniques such as PSMA-PET and genomic profiling, limitations remain in sensitivity, accessibility, and clinical integration, necessitating continued research into novel markers such as the centromere protein (CENPs) genes and microRNAs (miRNAs) for improved diagnostic precision.^4^^,^^5^

**Figure 1.**
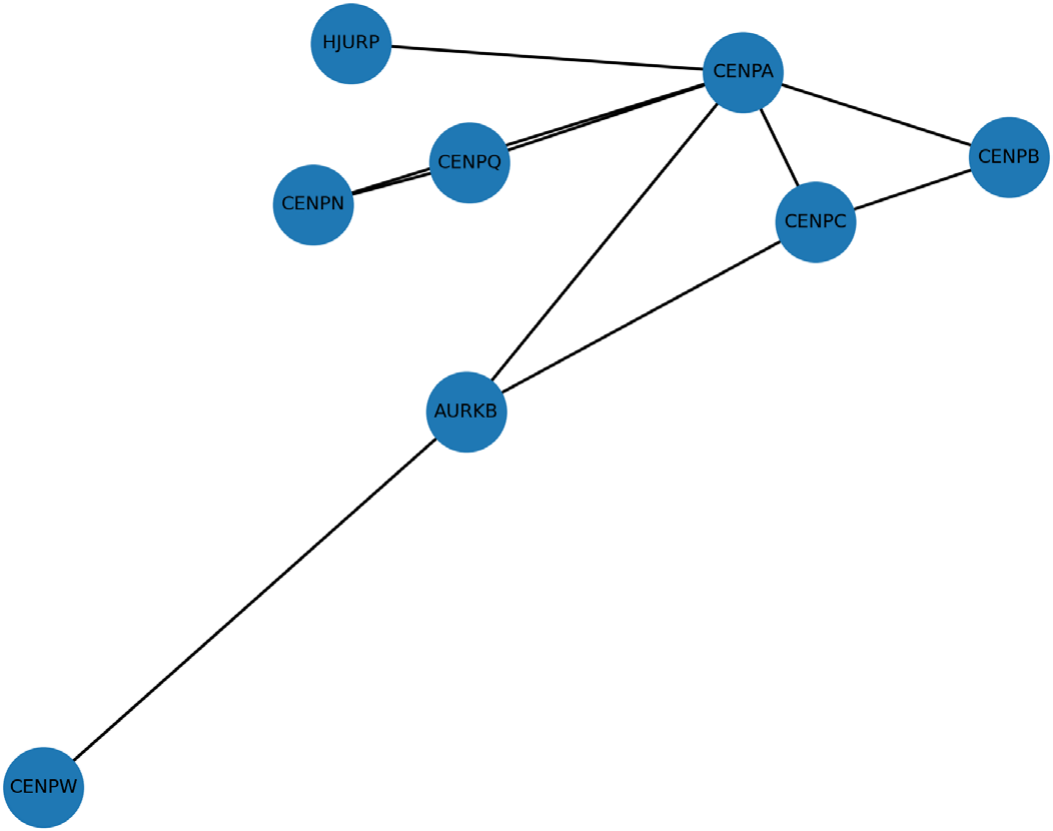
CENP genes signaling network.

CENP gene family plays a crucial role in maintaining chromosomal segregation and mitotic stability. Aberrations in their expression have been linked to oncogenesis through disruptions in genomic stability and cell cycle regulation. Simultaneously, miRNAs—short, non-coding RNAs—serve as post-transcriptional regulators and play dual roles as tumor suppressors or oncogenes depending on the cellular context. Notably, tumor-suppressive miRNAs can downregulate oncogenic targets involved in proliferation and metastasis, thereby inhibiting tumor progression.^6^ Conversely, downregulation or deletion of tumor-suppressive miRNAs, frequently observed in various malignancies, can release oncogenic pathways from repression, facilitating tumor growth and resistance mechanisms.^7^^,^^8^ This regulatory axis between miRNAs and CENP-related pathways presents a promising avenue for the identification of novel biomarkers and the development of targeted therapeutic strategies in aggressive malignancies. Therefore, the study aim to evaluate the expression patterns of CENPs genes (AURKB, CENPA, CENPC, CENPN, CENPQ, CENPW, HJURP)(Figure 1) and their regulatory miRNAs (hsa-miR-4755-5p, hsa-miR-5688, hsa-miR-4643, hsa-miR-3973, hsa-miR-20a-5p, hsa-miR-3941, hsa-miR-5680, hsa-miR-6825, hsa-miR-4500-5p) (Table 1) in PCa, with the goal of elucidating their potential roles in tumor progression and identifying novel molecular biomarkers for improved diagnosis and therapeutic targeting.

## Material and Methods

### Study Design and Reporting Standards

This study was conducted as a retrospective analysis of previously collected formalin-fixed paraffin-embedded (FFPE) prostate tissue samples from patients between 2018 and 2023 that were included in the study. The prostate cancer cell line was cultured under long-term standard culture conditions and serially passaged for 3 months. To confirm the stability of gene expression findings, CENPs gene expression analyses were performed on PCa cell line samples collected at 2 independent time points. The manuscript was prepared and reported in full compliance with the Strengthening the Reporting of Observational Studies in Epidemiology (STROBE) guidelines.

### Study Setting and Ethical Approval

The study was conducted at Necmettin Erbakan University, Faculty of Medicine, Medical Genetics Laboratory. Ethical approval was obtained from the Ethics Committee for Non-Drug and Non-Medical Device Research of Necmettin Erbakan University (Approval no: 2023/4129, Date: January 6, 2023).

All procedures were performed in accordance with the Declaration of Helsinki. Written informed consent was obtained from each participant in accordance with ethical standards prior to study inclusion.

### Study Population and Sample Selection

#### Patient Groups:

Formalin-fixed paraffin-embedded prostate tissue samples were retrospectively collected from patients who underwent diagnostic or therapeutic procedures in the Department of Urology. A total of 65 individuals were included and categorized into 3 groups:

1. Control group: Patients diagnosed with benign prostatic hyperplasia (n = 31).
2. Localized prostate cancer group: Patients with histopathologically confirmed localized prostate cancer (n = 20).
3. Metastatic prostate cancer group: Patients with radiologically and/or clinically confirmed metastatic prostate cancer (n = 14).

### Inclusion Criteria

Availability of adequate FFPE tissue material, histopathological confirmation of diagnosis, no history of other malignancies, and provision of written informed consent are the ınclusion criteria of this research.

### Exclusion Criteria

Insufficient or degraded tissue material, prior chemotherapy or radiotherapy before tissue sampling, presence of concomitant malignant diseases, clinicopathological data, including Gleason score and metastatic status**,** were retrieved from medical records.

### Cell Line and In Vitro Experimental Design

The metastatic prostate cancer cell line PC3 was cultured under standard long-term culture conditions in a humidified incubator containing 5% CO_2_ at 37°C (Sanyo Panasonic MCO-20AIC). Cells were monitored regularly using an inverted microscope. RNA samples were collected at 2 predefined time points to evaluate temporal changes in gene expression.

### microRNA Selection

In order to identify the genes in the targeted CENPs gene pathway, the associated miRNAs were first scored from the “MiRBASE and MiRDB” databases. Those with the highest scores were then selected from the miRTissue atlas database, on the basis of their expression in human prostate tissue (Supplementary Table 1).

### RNA and microRNA Isolation

#### Cell Line Samples:

Total RNA was extracted from cultured PC3 cells using a commercial RNA isolation kit (Qiagen, Cat. No. 217504) in accordance with the manufacturer’s protocol. RNA quantity and purity were determined by NanoDrop spectrophotometric analysis.

#### Formalin-Fixed Paraffin-Embedded Tissue Samples:

Formalin-fixed paraffin-embedded tissue blocks were processed in the Department of Pathology. Deparaffinization was performed using xylene, followed by ethanol washes. Total RNA and microRNA were extracted using the miRNeasy FFPE Kit and SanPrep Column miRNA Mini-Preps Kit (Bio Basic, Cat. No. SK8811-50PREPS). RNA quantity and quality were evaluated using a NanoDrop 2000 spectrophotometer (Thermo Fisher Scientific, USA). Samples with an A260/A280 ratio ≥1.7 were included. Extracted RNA was stored at −80°C until further analysis.

#### cDNA Synthesis:

cDNA synthesis for microRNA expression analysis was performed using the miRNA All-In-One cDNA Synthesis Kit (ABM, Cat. No. G898) as per the manufacturer’s protocol.

#### Quantitative Real-Time PCR (qRT-PCR):

Gene and microRNA expression analyses were conducted by quantitative real-time PCR (qRT-PCR) on the LightCycler 480 System (Roche) with FastStart Essential DNA Green Master Mix (Roche, Cat. No. 06402712001). Target genes included (AURKB, CENPA, CENPB, CENPC, CENPN, CENPQ, CENPW, HJURP), along with selected regulatory microRNAs. Primer specificity and amplification efficiency were validated prior to analysis, with efficiencies ranging between 90% and 110%.

All reactions were performed in triplicate. No-template controls and no reverse transcription controls were included in each run, and no nonspecific amplification was observed.

#### Reference Genes and Normalization:

For microRNA expression analysis, RNU6 was used as the endogenous reference gene. For mRNA expression analysis, GAPDH was selected as the reference gene. These reference genes were chosen based on their stable expression across all experimental and clinical groups.

#### Expression Level Calculation:

Cycle threshold (Ct) values were obtained automatically. Relative expression levels were calculated using the 2^−^ΔCt and 2^−^ΔΔCt methods, where: ΔCt = Ct_target − Ct_reference; ΔΔCt = ΔCt_sample − ΔCt_control; fold change values were calculated as 2^−^ΔΔCt.

### Statistical Analysis

Statistical analyses were performed using SPSS for Windows (version 21.0; IBM Corp., Armonk, NY, USA) and Jamovi (version 2.6.26). Data distribution was assessed using the Shapiro–Wilk test. Normally distributed variables were analyzed using Student’s *t*-test or 1-way analysis of variance, followed by Tukey’s post hoc test. Non-normally distributed variables were analyzed using the Mann–Whitney *U*-test or Kruskal–Wallis test. Categorical variables were compared using the chi-square test. All statistical tests were 2-sided, and a *P* value < .05 was considered statistically significant.

## Results

**Figure 2.**
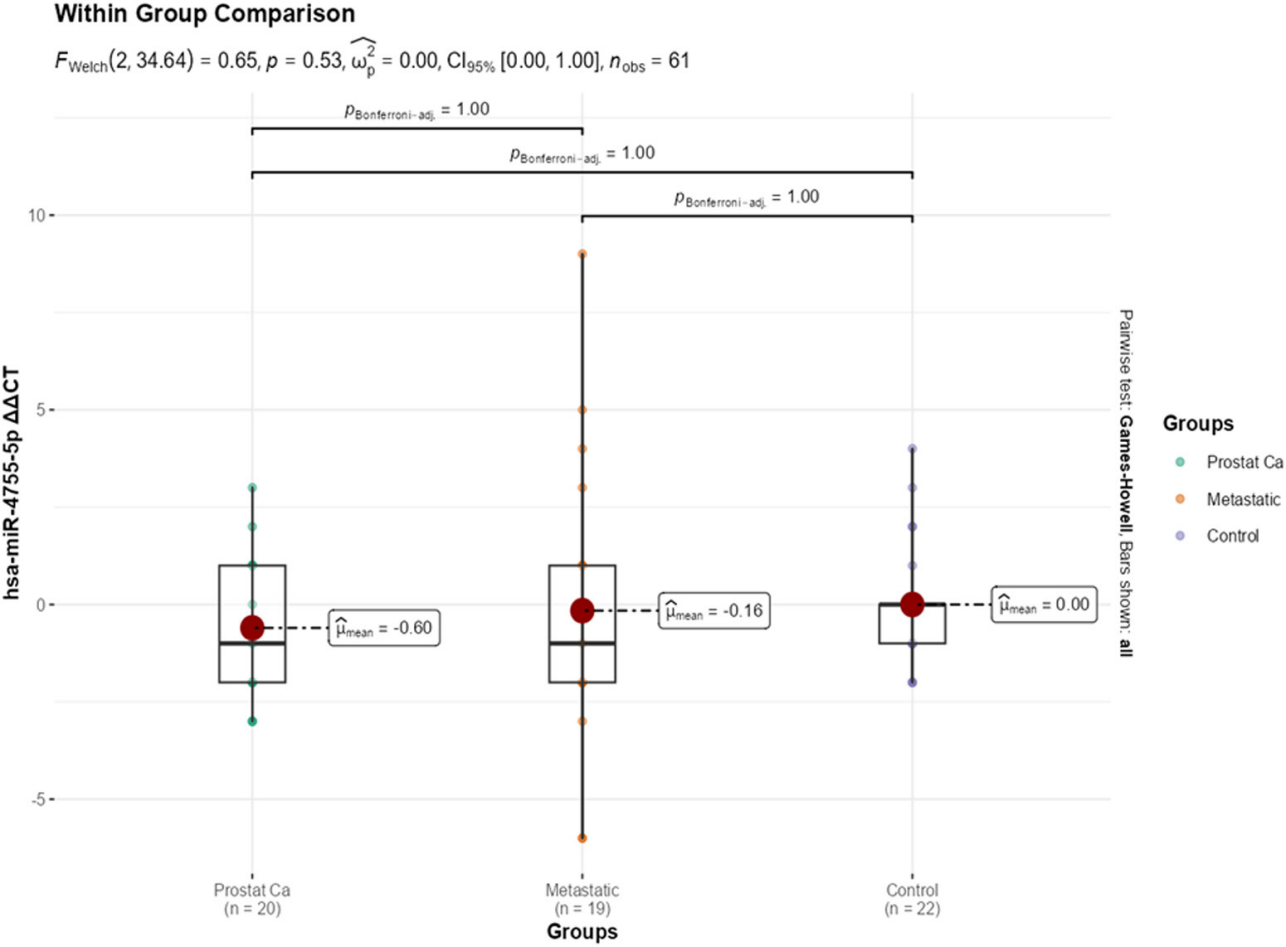
Group-wise comparison (*P* < .05) of ΔΔCT for hsa-miR-4755-5p.

**Figure 3.**
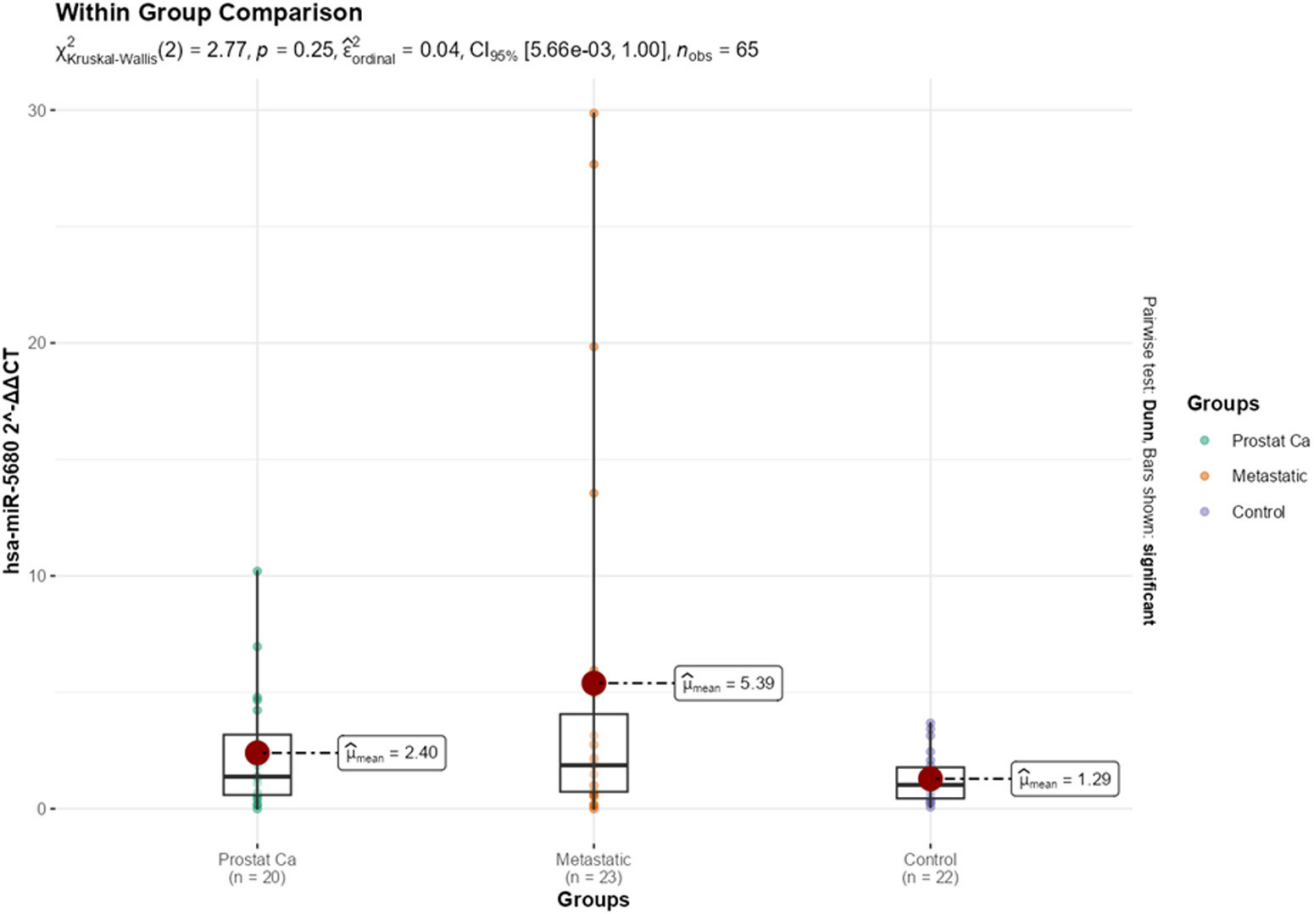
Group-wise comparison of (*P* < .05) fold change for hsa-miR-5680.

The expression levels of miRNAs were evaluated across prostate cancer, metastatic prostate cancer, and control groups, revealing the following findings: as expected for a housekeeping gene, U6 Ct values showed no statistically significant difference across the prostate cancer, metastatic, and control groups (*P* = .795), supporting the reliability of normalization in this study. For hsa-miR-4755-5p, while Ct, ∆Ct, and fold change values did not differ significantly between groups (*P* > .05), the ∆∆Ct value in the metastatic group (−1.54 ± 2.66) was significantly lower than in the control group (0.50 ± 2.39), indicating an upregulation of miR-4755-5p expression in the metastatic group (Figure 2). Regarding hsa-miR-5680, although Ct, ∆Ct, and ∆∆Ct values did not significantly vary (*P* > .05), the fold change in the metastatic group (7.29 ± 10.16) was found to be statistically significantly higher compared to the control group (1.86 ± 3.48) (*P* = .034) (Figure 3). For the remaining miRNAs including hsa-miR-5688, hsa-miR-4643, hsa-miR-3973, hsa-miR-20a-5p, hsa-miR-4500, and hsa-miR-6825-5p no statistically significant differences were observed between the groups in terms of their Ct, ∆Ct, ∆∆Ct, or fold change values (*P* > .05 for all). Therefore, these miRNAs did not demonstrate differential expression across the studied groups (Table 1).

To observe cell cycle control gene expression regulated by miRNAs in prostate cancer cells, the PCa cell line was cultured for 2 weeks. Cycle threshold values were normalized to GAPDH to obtain ΔCT values, and relative gene expression was quantified using the comparative Ct (2^−ΔΔCt) method. The comparative analysis revealed that HJURP, CENPC, and CENPA were upregulated at the second time point, while AURKB was downregulated. Other genes such as CENPQ, CENPN, andCENPW lacked second time point measurements and were reported descriptively (Table 2).

GAPDH was employed as a housekeeping gene to ensure reliable normalization and eliminate technical variability, reinforcing the validity of comparative gene expression results (GAPDH-1 CT: 16.24; GAPDH-2 CT: 20.83) (Table 2).

### Summary of Key Findings

Collectively, these results demonstrate that metastatic prostate cancer is characterized by distinct dysregulation of specific microRNAs, including significant upregulation of hsa-miR-4755-5p and hsa-miR-5680, together with a non-significant but consistent downregulation trend of hsa-miR-5688, hsa-miR-20a-5p, and hsa-miR-4500, combined with altered expression of centromere-associated genes. These molecular alterations may contribute to tumor progression and metastatic behavior.

## Discussion

Tumor-suppressive miRNAs function by repressing oncogenic transcripts involved in proliferation, invasion, and survival pathways, whereas loss or reduction of these miRNAs may facilitate tumor progression.^10^^,^^11^ In the present study, hsa-miR-4755-5p and hsa-miR-5680 were significantly upregulated in metastatic prostate cancer, while hsa-miR-5688, hsa-miR-20a-5p, and hsa-miR-4500 exhibited a consistent but non-significant trend toward downregulation. In published data, direct studies on hsa-miR-5680 and hsa-miR-4755-5p in PCa remain limited; these miRNAs are rarely featured in prostate cancer-focused datasets or validation studies. Furthermore, an increasing body of research has focused on elucidating the regulatory mechanisms of miRNAs in various malignancies, given their dual role in either promoting or suppressing tumor progression. Recently, Zang et al investigated the molecular basis of lymph node metastasis in gastric cancer and revealed a novel MIR181A2HG/miR-5680/VCAN-CD44 regulatory axis that facilitates immune modulation and metastatic progression. In contrast, the finding of increased hsa-miR-5680 expression in metastatic prostate cancer may reflect a compensatory or context-dependent regulatory response, highlighting that miRNA function and directionality can vary substantially across cancer types. Similar observations have been reported in hepatocellular carcinoma, where altered miR-5680 expression has been associated with survival outcomes.^13^ MicroRNAs are also increasingly recognized as essential mediators of chemoresistance, capable of modulating gene expression post-transcriptionally and impacting cancer cell survival and treatment response. hsa-miR-4755-5p has been previously implicated in cancer progression and therapy resistance. Lei et al^14^ reported that miR-4755-5p contributes to decitabine resistance in hematologic malignancies by targetingCDKN2B, thereby promoting cell survival and proliferation. Consistent with these findings, the significant upregulation of hsa-miR-4755-5p observed in metastatic prostate cancer in the study suggests a potential role in advanced disease biology, although functional validation in solid tumors is warranted. On the other hand, functional annotations or validated targets for miRNAs in PCa are lacking; similar upregulations of novel or less-characterized miRNAs have been reported in aggressive cancer phenotypes, possibly reflecting secondary transcriptional changes or adaptive regulatory shifts. Xu et al^15^ identified a range of novel miRNAs in cancer-associated fibroblasts that promoted tumor growth and resistance in PCa, suggesting that previously understudied miRNAs can play impactful roles in tumor microenvironments. In addition, Aldakheel et al^16^ identified multiple dysregulated miRNAs influencing transcriptomic patterns in PCa. Similarly, other high-throughput screenings, such as those by Palanisamy et al,^17^ emphasized miR-133b and miR-17-5p as strong prognostic indicators in PCa. Given the limited literature, the upregulation of hsa-miR-4755-5p and hsa-miR-5680 in the metastatic prostate cancer group may reflect a novel or unrecognized regulatory shift in advanced disease. This could indicate a compensatory response or dysregulated pathway, warranting further validation in larger cohorts and functional studies.

Recent studies highlight the diagnostic potential of miRNA combinations over single markers in prostate cancer. Osip’yants et al conducted a case-control study of 36 patients (18 metastatic, 18 non-metastatic) and found that while individual circulating miRNAs lacked diagnostic significance, specific pairs such as hsa-miR-19b-3p and hsa-miR-297 showed high sensitivity and specificity. This pair commonly targets CFL2, a gene involved in cytoskeletal remodeling and linked to cancer cell invasiveness. The authors suggest that downregulation of these miRNAs may upregulate CFL2, facilitating metastasis.^18^ Although not reaching statistical significance, hsa-miR-5688, hsa-miR-20a-5p, and hsa-miR-4500 showed a consistent trend toward downregulation in both localized and metastatic prostate cancer. Similar downregulation patterns of poorly characterized miRNAs have been reported in aggressive cancer phenotypes and may reflect loss of tumor-suppressive regulatory mechanisms.^19-^^21^

miR-20a-5p, in particular, has been reported to exhibit dual oncogenic or tumor-suppressive roles depending on cellular context.^22^^-^^25^ The downregulation trend observed in the cohort may represent a context-specific response in prostate cancer progression, further underscoring the complexity of miRNA-mediated regulation. In vitro analysis demonstrated increased expression of HJURP, CENPA, andCENPC,alongside downregulation ofAURKB in the PC3 prostate cancer cell line over time. These findings are largely consistent with existing literature linking centromere-associated proteins to chromosomal instability and tumor progression.^26^^-^^28^

Although AURKB is frequently reported as overexpressed in aggressive prostate cancer, the observed downregulation in the PC3 cell line may reflect cell line-specific or temporal regulatory dynamics. Since AURKB expression was evaluated only in vitro, these results should be interpreted cautiously and may not fully represent clinical tissue behavior.^29^

In the study, the gene expression data from the study, showing upregulation of HJURP, CENPA, and CENPC, and downregulation of AURKB at the second timepoint in a prostate cancer cell line, are largely consistent with the emerging literature underscoring the oncogenic potential of centromere-associated genes. The discrepancy in AURKB expression may reflect tumor heterogeneity or cell line-specific dynamics and highlights the complexity of mitotic regulation in prostate cancer biology. The gene expression data aligns with current evidence linking centromere-associated proteins and mitotic regulators with cancer progression and may highlight potential targets for therapeutic intervention in PCa.

### Study Limitations

Certain limitations should be acknowledged. The sample size, while sufficient to demonstrate significant expression differences, limits subgroup analyses. Additionally, functional validation experiments were beyond the scope of this study. Nevertheless, the integration of clinical samples with in vitro validation represents a notable strength and supports the robustness of the findings.

## Conclusion

The findings underscore the emerging significance of both novel and established microRNAs, as well as centromere-associated genes, in the progression and metastatic behavior of PCa. The upregulation of hsa-miR-4755-5p and hsa-miR-5680 in metastatic samples may represent compensatory or deregulated responses associated with advanced disease, while the consistent downregulation trends of tumor-suppressive miRNAs such as hsa-miR-5688, hsa-miR-20a-5p, and hsa-miR-4500 suggest a loss of regulatory control that could facilitate oncogenic signaling. Additionally, the increased expression of HJURP, CENPA, and CENPC, alongside AURKB downregulation, points to the complex and temporally dynamic nature of mitotic regulation in PCa. Ultimately, these findings underscore the importance of integrating emerging miRNA signatures and cell cycle-related gene networks into future biomarker development efforts, and warrant validation in larger, longitudinal patient cohorts to better understand their diagnostic, tumor-regulator, and therapeutic potential in PCa.

In the present study, a distinct molecular signature in metastatic prostate cancer characterized by the coordinated dysregulation of selected microRNAs and centromere-associated cell cycle genes was demonstrated. While previous studies have independently reported alterations in either microRNA expression or mitotic regulatory genes in prostate cancer, the findings provide integrated evidence linking these molecular events to metastatic progression.

### Clinical Relevance and Implications

From a clinical perspective, the identification of reliable biomarkers capable of distinguishing aggressive or metastatic prostate cancer from localized disease remains a major challenge in urologic oncology. The observed upregulation of hsa-miR-4755-5p and hsa-miR-5680, together with the downregulation trend of hsa-miR-5688, hsa-miR-20a-5p, and hsa-miR-4500, suggests that specific miRNA expression patterns may serve as potential molecular indicators of disease progression.

Importantly, the concomitant dysregulation of centromere-associated genes, including CENPA,CENPC, HJURP,and AURKB, supports a biological link between altered microRNA regulation and enhanced proliferative and metastatic capacity. Collectively, these findings indicate that a combined miRNA–gene expression profile may improve risk stratification in prostate cancer, although prospective validation in larger cohorts is required.

## Figures and Tables

**Table 1.** Comparison of microRNA (miRNA) Expression Levels (Ct, ∆Ct, ∆∆Ct, and Fold Change) Among the Groups

	**Prostate Ca (n = 20) (Mean ± SD)**	**Metastatic Prostate (n = 14) (Mean ± SD)**	**Control Group (n = 31) (Mean ± SD)**	***P***
U6 Ct	23.62 ± 1.33	23.90 ± 1.32	23.81 ± 1.13	.795
hsa-miR-4755-5p Ct ∆Ct ∆∆Ct Fold change	33.39 ± 1.19 9.76 ± 1.72 −0.57 ± 1.72 2.66 ± 2.73	33.90 ± 3.35 13.22 ± 11.54 −1.54 ± 2.66 10.00 ± 19.05	34.61 ± 2.20 10.45 ± 8.95 0.50 ± 2.39 1.42 ± 1.15	.206 .273 .027* **^a^** .122
hsa-miR-5688 Ct ∆Ct ∆∆Ct Fold change	37.06 ± 2.06 13.43 ± 2.44 0.25 ± 2.44 2.27 ± 2.80	35.88 ± 1.60 11.76 ± 1.98 −1.54 ± 2.02 6.15 ± 11.27	37.06 ± 2.11 10.77 ± 9.35 −0.02 ± 2.55 4.82 ± 16.60	.344 .246 .130 .224
hsa-miR-4643 Ct ∆Ct ∆∆Ct Fold change	38.63 ± 2.84 15.00 ± 3.54 −0.56 ± 3.54 6.58 ± 7.89	37.33 ± 2.39 10.09 ± 11.35 −2.30 ± 3.02 16.08 ± 29.16	38.92 ± 2.70 14.62 ± 9.37 −0.79 ± 2.65 6.29 ± 13.47	.167 .215 .285 .502
hsa-miR-3973 Ct ∆Ct ∆∆Ct Fold change	35.98 ± 1.37 5.15 ± 14.50 −0.002 ± 1.58 1.54 ± 1.48	35.21 ± 2.36 8.15 ± 10.58 −1.08 ± 2.63 5.58 ± 8.97	36.27 ± 1.26 8.55 ± 13.38 0.05 ± 1.51 1.52 ± 2.21	.180 .495 .204 .543
hsa-miR-20a-5p Ct ∆Ct ∆∆Ct Fold change	34.72 ± 1.84 11.09 ± 2.52 0.23 ± 2.5 2.03 ± 2.34	34.99 ± 2.57 12.71 ± 9.02 −0.59 ± 1.91 2.95 ± 3.96	35.33 ± 1.99 12.29 ± 5.90 0.43 ± 2.03 2.52 ± 8.15	.585 .410 .388 .360
hsa-miR-4500 Ct ∆Ct ∆∆Ct Fold change	39.68 ± 2.28 8.12 ± 15.99 −0.06 ± 1.91 1.76 ± 1.84	38.51 ± 2.59 11.17 ± 11.65 −1.49 ± 2.89 7.62 ± 12.65	39.89 ± 3.18 4.11 ± 18.65 0.03 ± 3.64 4.77 ± 9.65	.395 .625 .363 .520
hsa-miR-5680 Ct ∆Ct ∆∆Ct Fold change	35.18 ± 2.25 11.56 ± 3.08 0.04 ± 3.08 2.53 ± 2.63	34.85 ± 2.89 12.19 ± 9.03 0.67 ± 9.03 7.29 ± 10.16	35.47 ± 1.29 11.62 ± 1.68 0.10 ± 1.74 1.86 ± 3.48	.137 .095 .104 .034* **^b^**
hsa-miR-6825 Ct ∆Ct ∆∆Ct Fold change	36.04 ± 1.93 5.20 ± 15.16 −0.22 ± 2.62 2.48 ± 2.61	35.12 ± 2.23 5.13 ± 14.09 −1.63 ± 2.57 6.10 ± 10.12	36.65 ± 1.53 5.51 ± 14.76 −0.01 ± 1.91 2.43 ± 6,59	.088 .261 .172 .098

*P < .05 statistically significant.

^a^Significantly lower ΔΔCt in metastatic vs. control group (*P* < .05, post hoc – Tukey test).

^b^2^-ΔΔCt significantly higher in metastatic > PCa and control groups (*P* < .05, post hoc – Tukey test).

**Table 2.** Normalized Gene Expression Values (CT, ΔCT, ΔΔCT, and 2^−ΔΔCT) of Selected Genes at 2 Time Points in a Prostate Cancer Cell Line

**Gene**	**Sample**	**CT**	**ΔCT**	**ΔΔCT**	**2^−ΔΔCT**	**Interpretation**
*HJURP*	* HJURP-2 *	27.55 27.74	11.31 6.91	0.00 −4.40	1.000 21.112	Baseline Upregulated
*AURKB*	* AURKB-2 *	23.93 31.44	7.69 10.61	−3.62 −0.70	12.295 1.624	Baseline Downregulated
*CENPA*	* CENPA-2 *	34.37 33.79	18.13 12.96	6.82 1.65	0.0089 0.3186	Baseline Upregulated
*CENPC*	* CENPC-2 *	34.25 36.93	18.01 16.10	6.70 4.79	0.0096 0.0361	Baseline Upregulated
*CENPQ*		36.76	20.52	9.21	0.0017	No data
*CENPN *		37.64	21.40	10.09	0.0009	No data
*CENPW *		35.15	18.91	7.60	0.0052	No data

**Supplementary Table 1.** Forward and reverse primer sequences (5’–3’) used for gene expression analysis by qPCR

Gene	Forward Primer (5'-3')	Reverse Primer (5'-3')
ACTB	CTTCGCGGGCGACGAT	CCACATAGGAATCCTTCTGACC
GAPDH	GGAGCGAGATCCCTCCAAAAT	GGCTGTTGTCATACTTCTCATGG
CENPA	TGGCTAAAGGAGATCCGAAAG	GCATGTAAGGTGAGGAGATAGG
CENPN	AGCATCTTGGCTGAGAGGGA	ACTGTGGCTTCCAGAATAGCA
CENPW	AGCGAGTCTTCAAGCGAAAG	GCCAGTACATGCTCCTTGTTA
CENPQ	GGACAAACAAAGCACACTAACC	CCTGTAGTAATGCCAGACCTTC
CENPC	GTGCCAGAAATCACATCCAAAG	AAGGTGAGCCAACGGATAAG
AURKB	TTATGACCGGAGGAGGATCTAC	CAATCTTCAGCTCTCCCTTGAG
HJURP	CCCAAGAGCGATTCATCTTCAT	CCCAGGAGATTTGAGGCAATAC

## Data Availability

The data that support the findings of this study are available on request from the corresponding author.
